# Towards Generating and Evaluating Iconographic Image Captions of Artworks

**DOI:** 10.3390/jimaging7080123

**Published:** 2021-07-23

**Authors:** Eva Cetinic

**Affiliations:** 1 Rudjer Boskovic Insitute, Bijenicka Cesta 54, 10000 Zagreb, Croatia; ecetinic@irb.hr; 2 Department of Computer Science, Durham University, Durham DH1 3LE, UK

**Keywords:** image captioning, vision-language models, fine-tuning, visual art

## Abstract

To automatically generate accurate and meaningful textual descriptions of images is an ongoing research challenge. Recently, a lot of progress has been made by adopting multimodal deep learning approaches for integrating vision and language. However, the task of developing image captioning models is most commonly addressed using datasets of natural images, while not many contributions have been made in the domain of artwork images. One of the main reasons for that is the lack of large-scale art datasets of adequate image-text pairs. Another reason is the fact that generating accurate descriptions of artwork images is particularly challenging because descriptions of artworks are more complex and can include multiple levels of interpretation. It is therefore also especially difficult to effectively evaluate generated captions of artwork images. The aim of this work is to address some of those challenges by utilizing a large-scale dataset of artwork images annotated with concepts from the Iconclass classification system. Using this dataset, a captioning model is developed by fine-tuning a transformer-based vision-language pretrained model. Due to the complex relations between image and text pairs in the domain of artwork images, the generated captions are evaluated using several quantitative and qualitative approaches. The performance is assessed using standard image captioning metrics and a recently introduced reference-free metric. The quality of the generated captions and the model’s capacity to generalize to new data is explored by employing the model to another art dataset to compare the relation between commonly generated captions and the genre of artworks. The overall results suggest that the model can generate meaningful captions that indicate a stronger relevance to the art historical context, particularly in comparison to captions obtained from models trained only on natural image datasets.

## 1. Introduction

Image captioning refers to the task of generating a short text that describes the content of an image based only on the image input. This usually implies recognizing objects and their relationships in an image. Those descriptions should be meaningful and accurate in relation to the image content. In resolving this task, significant progress has recently been made using multimodal deep learning models. However, most of the research in this field is performed on datasets of natural images, while the specific aspects of generating captions for artwork images have not yet been systematically explored.

A common prerequisite for training deep neural captioning models are large datasets of semantically related image and sentence pairs. In the domain of natural images, several well-known large-scale datasets are commonly used for this task, such as the MS COCO [1], Flickr30 [2] and Visual Genome [3] dataset. The availability of such large datasets enabled the development of image captioning models that achieve impressive results in generating high quality captions for photographs of various objects and scenes. However, the task of generating image captions still remains difficult for domain-specific image collections. In particular, in the context of visual art and cultural heritage, generating image captions is an open problem with various challenges. The lack of a truly large-scale dataset of artwork images paired with adequate descriptions represents one of the major difficulties. Furthermore, it is important to address what kind of description would be regarded as “adequate” in the context of art historical data collections. Taking into account Erwin Panofsky’s three levels of analysis [4], we can distinguish the “pre-iconographic” description, “iconographic” description and the “iconologic” interpretation as possibilities of aligning meaningful, yet very different textual descriptions with the same image. Image captioning in the context of natural images is usually performed at the level of “pre-iconographic” descriptions, which implies simply describing the content and listing the objects that are depicted in an image. For artwork images this type of description represents only the most basic level of visual understanding and is not considered to be particularly useful for performing multimodal analysis and retrieval within art collections.

A more interesting, as well as more challenging, task would be to generate “iconographic” captions that describe the contextual aspect of the subject matter. Creating a dataset for such a complex task is difficult because it requires expert knowledge in the process of collecting sentence-based descriptions of images. Several such art datasets of image-text pairs exist, but those mostly consist of only a few thousand examples and are therefore not suitable for training deep neural network models in the current state-of-the-art setting for image captioning. However, there are several existing large-scale artwork collections that associate images with textual descriptions in the form of keywords and specific concepts. In particular, a large-scale artwork dataset, published under the name “Iconclass AI Test Set” [5], represents a collection of various artwork images assigned with alphanumeric classification codes that correspond to notations from the Iconclass system [6]. Iconclass is a classification system designed for art and iconography and is widely accepted by museums and art institutions as a tool for the description and retrieval of subjects represented in images. The idea of this work is to use a concatenation of the various code descriptions associated with an image as textual inputs for training an image captioning model. Although the “Iconclass AI Test Set” is not structured primarily as an image captioning dataset, each code is paired with its “textual correlate”—a description of the iconographic subject of the particular Iconclass notation. The first methodological step of the approach presented in this work includes extracting and preprocessing the given annotations into clean textual description and creating the “Iconclass Caption” dataset. This dataset is then used to fine-tune a pretrained unified vision-language model on the down-stream task of image captioning [7]. Transformer-based vision-language pretrained models currently represent the leading approach in solving a variety of tasks in the intersection of computer vision and natural language processing.

The work presented in this paper is an extension of a previous work that represents one of the first attempts in generating captions for artworks [8]. The methodological approach is similar and the additional contribution of this paper is primarily focused on the problem of evaluating the generated image captions. The previous work showed that standard reference-based image metrics are not very suitable for assessing the quality of image captions because they take into account only the relation between the generated and ground-truth caption, and not the relation between the caption and the image itself, which is particularly important in the context of artworks. Recently, significant advances have been achieved in transforming image and text embeddings into a joint feature space. Based on those findings, this work additionally explores how CLIP (Contrastive Language-Image Pre-training), a newly introduced cross-modal model pretrained on very large dataset of 400 M image+text pairs extracted from the web [9], and reference-free captioning metrics defined based on CLIP features [10], can be used to evaluate the generated iconographic captions.

## 2. Related Work

The availability of large collections of digitized artwork images fostered research initiatives in the intersection of artificial intelligence and art history. Most commonly, research in this area focuses on addressing problems related to computer vision in the context of art data, such as image classification [11,12,13], visual link retrieval [14,15,16], object and face detection [17,18], pose and character matching [19,20], analysis of visual patterns and conceptual features [21,22,23,24], and computational aesthetics [25,26,27]. A comprehensive overview of research activities in this area can be found in several survey papers [28,29,30].

Recently, there has been a surge of interest in topics related to jointly exploring both visual and textual modalities of artwork collections. Pioneering works in this research area addressed the task of multimodal retrieval. In particular, Ref. [31] introduced the SemArt dataset, a collection of fine-art images associated with textual comments, with the aim to map the images and their descriptions in a joint semantic space. They compare different combinations of visual and textual encodings, as well as different methods of multimodal transformation. In projecting the visual and textual encodings in a common multimodal space, they achieve the best results by applying a neural network trained with cosine margine loss on ResNet50 features as visual encodings and bag of word as textual encodings. The task of creating a shared embedding space was also addressed in [32], where the authors introduce a new visual semantic dataset named BibleVSA, a collection of miniature illustrations and commentary text pairs, and explore supervised and semi-supervised approaches to learning cross-references between textual and visual information in documents. In [33], the authors present the Artpedia dataset, consisting of 2930 images annotated with visual and contextual sentences. They introduce a cross-modal retrieval model that projects images and sentences in a common embedding space and discriminates between contextual and visual sentences of the same image. A similar extension of this approach to other artistic datasets was presented in [34]. Recently, Banar et al. introduced a study that explores how Iconclass codes can be automatically assigned to visual artworks using a cross-modal retrieval set-up [35].

Apart from multimodal retrieval, another recently emerging topic of interest is visual question answering (VAQ). In [36], the authors annotated a subset of the ArtPedia dataset with visual and contextual question–answer pairs and introduced a question classifier that discriminates between visual and contextual questions and a model that is able to answer both types of questions. In [37], the authors introduce a novel dataset AQUA (Art QUestion Answering), which consists of automatically generated visual and knowledge-based question-answer pairs, and also present a two-branch model where the visual and knowledge questions are handled independently.

The task of image captioning has not been significantly studied in the context of art images. A limited number of studies contributed to the task of generating descriptions of artwork images using deep neural networks. For example, Ref. [38] proposes an encoder–decoder framework for generating captions of artwork images where the encoder (ResNet18 model) extracts the input image feature representation and the artwork type representation, while the decoder is a long short-term memory (LSTM) network. They introduce two image captioning datasets referring to ancient Egyptian art and ancient Chinese art, which contain 17,940 and 7607 images, respectively. Another work [39] presented a novel captioning dataset for art historical images consisting of 4000 images across nine iconographies, along with a description for each image consisting of one or more paragraphs. They used this dataset to fine-tune different variations of image captioning models based on the well-known encoder–decoder approach introduced in [40]. As already mentioned, this paper represent an extension of the image captioning approach presented in [8].

Motivated by the success of utilizing large-scale pretrained language models such as the BERT (Bidirectional Encoder Representations from Transformers) model [41] for different tasks related to natural language processing, recently significant research progress has been made by adopting transformer-based models for a variety of multimodal tasks. Transformer-based vision-language models are designed to learn joint representations that combine and align information from both modalities. It has been shown that models pretrained on intermediate tasks with unsupervised learning objectives using large datasets of image-text pairs achieve remarkable results when applied to different down-stream tasks such as image captioning, cross-modal retrieval or visual question answering [7,42,43,44]. Furthermore, recently an efficient method of learning from natural language supervision was introduced as the CLIP (Contrastive Language-Image Pre-training) model [9]. The model is a result of training an image and text encoder to predict the correct pairs of image-text training examples using large amounts of publicly available internet data. The CLIP model showed very promising results on a variety of image-text similarity estimation tasks and was recently introduced as a novel way of establishing a reference-free image captioning metric [10]. This paper explores how those newly introduced image captioning metrics, as well as CLIP image and text representations, can be used to evaluate captions in the context of artworks.

## 3. Methodology

### 3.1. Datasets

#### 3.1.1. Iconclass Caption Dataset for Training and Evaluation

The main dataset used in this work is the “Iconclass AI Test Set” [5] dataset. The dataset contains, in total, 87,749 images, and in this work 86,530 valid image-text pairs are used for training and evaluating the image captioning model (1219 images do not have valid codes/textual notations assigned to them). The dataset includes a very diverse collection of images sampled from the Arkyves database www.arkyves.org (accessed on 21 June 2021). It includes images of various types of artworks such as paintings, posters, drawings, prints, manuscripts pages, etc. Each image is associated with one or more codes linked to labels from the Iconclass classification system. The authors of the “Iconclass AI Test Set” provide a json file with the list of images and corresponding codes, as well as an Iconclass Python package to perform analysis and extract information from the assigned classification codes. To extract textual descriptions of images for the purpose of this work, the English textual descriptions of each code associated with an image are concatenated. Further preprocessing of the descriptions includes removing text in brackets and some recurrent uppercased dataset-specific codes. In this dataset, the text in brackets most commonly includes very specific named entities, which are considered a noisy input in the image captioning task. Therefore, when preprocessing the textual items, all the text in brackets is removed, even at the cost of sometimes removing useful information.

**Figure 1:**
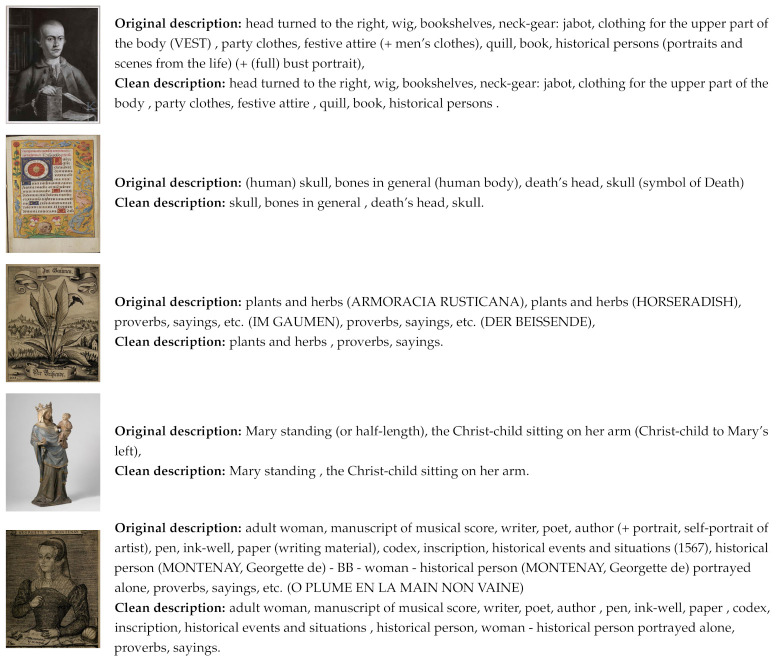
Example images from the Iconclass Caption dataset and their corresponding descriptions before and after preprocessing.

Figure 1 shows several example images from the Iconclass Caption dataset and their corresponding descriptions before and after preprocessing. Depending on the number of codes associated with each image, the final textual descriptions can significantly vary in length. Additionally, due to the specific properties of this dataset, the image descriptions are not structured as sentences but as a list of comma-separated words and phrases.

**Figure 2:**
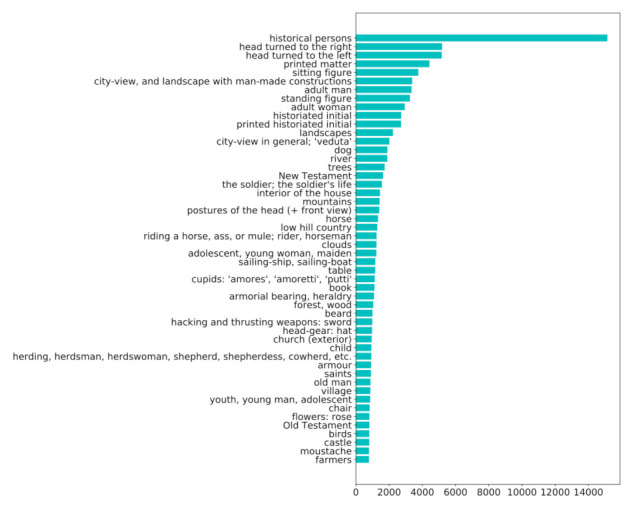
Distribution of textual descriptions in the Iconclass Caption dataset showing the 50 most commonly occurring words/phrases (Iconclass codes) in the whole dataset.

The textual descriptions are represented as a concatenation of text phrases related to the Iconclass codes. One image in the dataset can be associated with one or more textual phrases. To better understand the configuration of the dataset, Figure 2 shows a distribution of the most commonly included textual phrases (Iconclass codes).

Due to this type of structure and having only one reference caption for each image, the Iconclass Caption dataset is not a standard image captioning dataset. However, having in mind the difficulties of obtaining adequate textual descriptions for images of artworks, this dataset can be considered as a valuable source of image-text pairs in the current context, particularly due to the large number of annotated images that enables training deep neural models. In the experimental setting, a subset of approximately 76,000 items is used for training the model, while around 5000 items are used for validation and 5000 for testing.

#### 3.1.2. Wikiart Dataset for Evaluation

In order to explore how the proposed approach works on another artwork dataset, a subset of 52,562 images of paintings from the WikiArt, www.wikiart.org (accessed on 1 February 2020), collection was used. Images in the WikiArt dataset are annotated with a broad set of labels (e.g., style, genre, artist, technique, date of creation, etc.); therefore, one aspect of the evaluation process includes analysing how the generated captions relate to genre labels because genre labels indicate the category of the subject matter that is depicted (e.g., portrait, landscape, religious paintings, etc.). Furthermore, this dataset is used to explore the difference between captions generated using a model trained on artwork images and models trained on natural image datasets.

### 3.2. Image Captioning Model

For the purpose of training an image captioning model, in this work the unified vision-language pretraining model (VLP) introduced in [7] was employed. This model is denoted as “unified” because the same pretrained model can be fine-tuned for different types of tasks. These tasks include both vision-language generation (e.g., image captioning) and vision-language understanding (e.g., visual question answering). The model is based on an encoder–decoder architecture comprised of 12 transformer blocks. The model input consist of image embedding, text embedding and three special tokens that indicate the start of the image input, the boundary between the visual and textual input and the end of the textual input. The image input consists of 100 object classification aware region features extracted using the Faster R-CNN (region-based convolutional neural networks) model [45] pretrained on the Visual Genome dataset [3]. For a more detailed description of the overall VLP framework and pretraining objectives, the reader is referred to [7]. The experiments introduced in this work employ, as the base model, the VLP model pretrained on the Conceptual Captions dataset [46] using the sequence-to-sequence objective. This base model is fine-tuned on the Iconclass Caption dataset using recommended fine-tuning configurations, namely training with a constant learning rate of 3e-5 for 30 epochs. The weights of the Iconclass fine-tuned model, together with the data used for training the model (image IDs and descriptions), are available here: https://github.com/EvaCet/Iconclass-image-captioning (accessed on 22 July 2021).

### 3.3. Evaluation of the Generated Captions

The evaluation of the model’s performance includes both quantitative and qualitative analyses of the generated captions. To quantitatively evaluate the generated captions, standard language evaluation metrics for image captioning and novel reference-free image captioning methods are used. The standard metrics include the four BLEU metrics [47], METEOR [48] ROUGE [49] and CIDEr [50]. BLUE, ROUGE and METEOR are metrics that originate from machine translation tasks, while CIDEr was specifically developed for image caption evaluation. The BLUE metrics represent n-gram precision scores multiplied by a brevity penalty factor to assess the length correspondence of candidate and reference sentences. ROUGE is a metric that measures the recall of n-grams and therefore rewards long sentences. Specifically, ROUGE-L measures the longest matching sequence of words between a pair of sentences. METEOR represents the harmonic mean of precision and recall of unigram matches between sentences and additionally includes synonyms and paraphrase matching. CIDEr measures the cosine similarity between TF-IDF weighted n-grams of the candidate and the reference sentences. The TF-IDF weighting of n-grams reduces the score of frequent n-grams and appoints higher scores to distinctive words.

As the standard image captioning metrics measure the relation between generated and original captions, they do not address the relation between the image itself and the generated caption. Although translating images and text in a joint semantic space has been an ongoing research topic, the recently introduced CLIP model [9] achieves significant performance improvements in assessing the similarity between image and text. Based on the advanced performance of this model, Hassel et al. [10] introduce a novel reference-free metric called CLIPScore, which, according to their study, achieves the highest correlation with human judgements and outperforms existing reference-based metrics. The CLIPscore represents a rescaled value (multiplied by factor of 2.5) of the cosine similarity between image and generated caption text embeddings obtained using the CLIP ViT-B/32 model for feature extraction. They also introduce a reference-augmented version of this score, the RefCLIPScore, which is computed as a harmonic mean of the CLIPScore and the maximal reference cosine similarity. Image captioning datasets usually include more than one reference sentence per image; however, the Iconclass Caption dataset includes only one reference description. Therefore, in this work, the RefCLIPScore is described as a harmonic mean between the rescaled cosine similiarity between the CLIP embeddings of the image and generated caption (the CLIPScore) and the value of the cosine similarity between the CLIP embeddings of the reference caption and generated caption.

## 4. Results and Discussion

### 4.1. Quantitative Results

The relation between the generated captions and the reference captions on the Iconclass Caption test set was evaluated using standard image captioning metrics. To evaluate the relation between the generated caption and the input image, the new CLIPScore metric was used, both in its original and reference-augmented versions. The results on the Iconclass Caption test set are presented in Table 1. The Iconclass Caption test set contains 5192 images, but the reported CLIP-S and RefCLIP-S values are calculated only on a subset of 4928 images where the generated captions are shorter than 76 tokens, together with tokens that indicate the end and beginning of the text sequence. This was carried out because the CLIP model, which serves as a basis for the CLIPScore metric, was trained with the maximal textual sequence length set at 76 tokens. As the Iconclass Caption dataset contains descriptions of various lengths, including very long ones, some of the generated captions are also long. In order to test the model on all the examples in the Iconclass Caption test set, an alternative version of the whole dataset was created where all image descriptions have been shortened in order to fit into the range of the maximal sequence length. As most of the descriptions consist of comma-separated concatenations of words and phrases, the shortening has been performed to keep only so many concatenated phrases to meet the 76 tokens limit. However, this shortening of the descriptions led to an overall deterioration of the captioning results in comparison with the results on the original, non-shortened dataset presented in Table 1 (the values of the metric scores on the alternative version of the dataset are: Bleu 1: 0.11; Bleu 2: 0.10; Bleu 3: 0.092; Bleu 4: 0.08; METEOR: 0.115; ROUGE-L: 0.302; CIDEr: 1.57; CLIP-S: 0.596; RefCLIP: 0.677). It was therefore decided to present and use the model trained on the original version of the dataset for further analysis and to report the CLIP-S and RefCLIP-S scores on a slightly smaller subset of the test set.

The current results cannot be compared with any other work because the experiments were performed on a new and syntactically and semantically different dataset. However, the quantitative evaluation results are included to serve as a benchmark for future work. In comparison with current state-of-the-art caption evaluation results on natural image datasets (e.g., BLEU4 ≈ 37 for MS COCO and ≈30 for Flickr30 datasets) [7,51], the BLEU scores are lower for the Iconclass dataset. A similar behaviour was also reported in another study addressing iconographic image captioning [39]. On the other hand, the CIDEr score is quite high in comparison to the one reported for natural image datasets (e.g., CIDEr ≈ 116 for MS COCO and ≈68 for Flickr30 dataset) [7,51]. To better understand how standard metrics relate to the novel metrics, Table 2 shows the Spearman’s rank correlation coefficient between the values of standard and novel captioning metrics on the Iconclass Caption test set.

**Figure 3:**
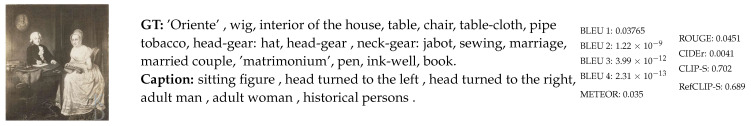

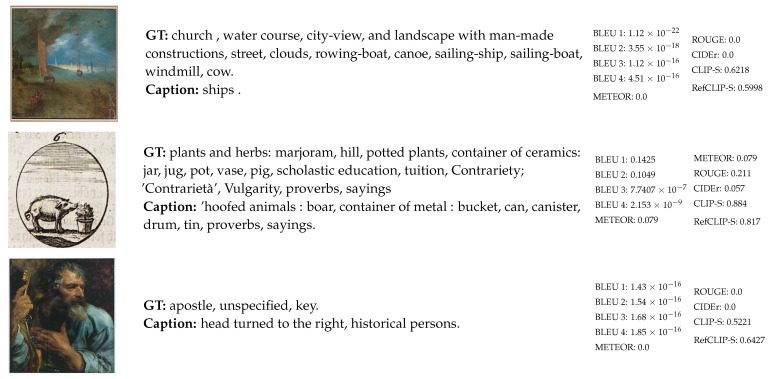
Examples of images from the Iconclass Caption test set, their corresponding ground-truth and generated captions and the values of evaluation metrics for those examples.

It is questionable how adequate standard reference-based metrics are in assessing the overall quality of the captions in this particular context because they mostly measure the word overlap between generated and reference captions. They are not designed to capture the semantic meaning of a sentence and therefore it is particularly difficult to evaluate iconographic descriptions. Furthermore, they are not appropriate for measuring very short descriptions which are quite common in the IconClass Caption dataset. Moreover, because they do not address the relation between the generated caption and the image content, the standard image captioning score could be low even if the generated caption is semantically aligned with the image content. In Figure 3, several such examples from the Iconclass Caption test set are presented, together with the values of the standard and new metrics.

In some examples within the Iconclass dataset, the generated caption is even more related to the image content than the ground-truth description (example image in row 3 in Figure 3) and interestingly the CLIP-Score is, in this case, higher than the usually higher RefCLIP-Score. Furthermore, those examples indicate that the standard evaluation metrics are not very suitable in assessing the relevance of generated captions for this particular dataset. Therefore, a qualitative analysis of the results is also required in order to better understand potential contributions and drawbacks of the proposed approach.

### 4.2. Qualitative Analysis

Qualitative analysis was performed by exploring examples of images and generated captions on two datasets. One is the test set of the Iconclass Caption dataset that serves for direct comparison between the generated captions and ground-truth descriptions. The other dataset is a subset of the WikiArt painting collection, which does not include textual descriptions of images but has a broad set of labels associated with each image. Therefore, this dataset is useful to explore how the generated captions relate to the genre categorization of the paintings.

#### 4.2.1. Iconclass Caption Test Set

**Figure 4:**
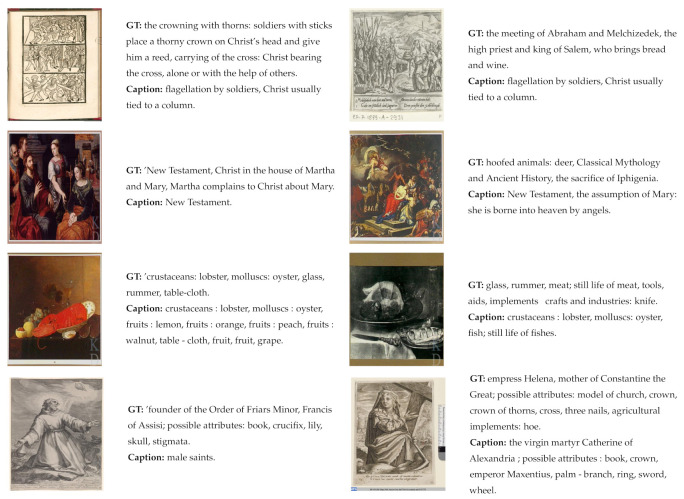
Examples of images from the Iconclass Caption test set, with their corresponding ground-truth and generated captions. Examples shown on the left side represent cases where the generated captions are successfully aligned with the iconographic content of the image, while examples shown on the right demonstrate unsuccessful examples.

To gain a better insight into the generated image captions, in Figure 4 several examples are shown. The presented image-text pairs were chosen to demonstrate both good examples (the left column) and bad examples (the right column) of generated captions.

Analysis of the unsuccessful examples indicates that similarities between visual representations can result in generating analogous, but very misleading, iconographic captions. It also demonstrates underlying biases within the dataset. For instance, in the Iconclass Caption training test, there are more than a thousand examples that include the phrase “New Testament” in the description. Therefore, images that include structurally similar scenes, particularly from classical history and mythology, are sometimes wrongly attributed as depicting a scene from the New Testament. This signifies the importance of balanced examples in the training dataset and indicates directions for possible future improvements. Furthermore, by analysing various examples of generated captions, it becomes clear that recognizing fine-grained categories, e.g., exact names of saints or specific historical scenes, is still a very challenging task.

The Iconclass dataset is a collection of very diverse images and apart from the Iconclass classification codes, there are currently no other metadata available for the images. Therefore, it is difficult to perform an in-depth exploratory analysis of the dataset and the generated results in regard to attributes relevant in the context of art history such as the date of creation, style, genre, etc. For this reason, the fine-tuned image captioning model was employed on another artwork dataset.

#### 4.2.2. WikiArt Dataset

**Figure 5:**
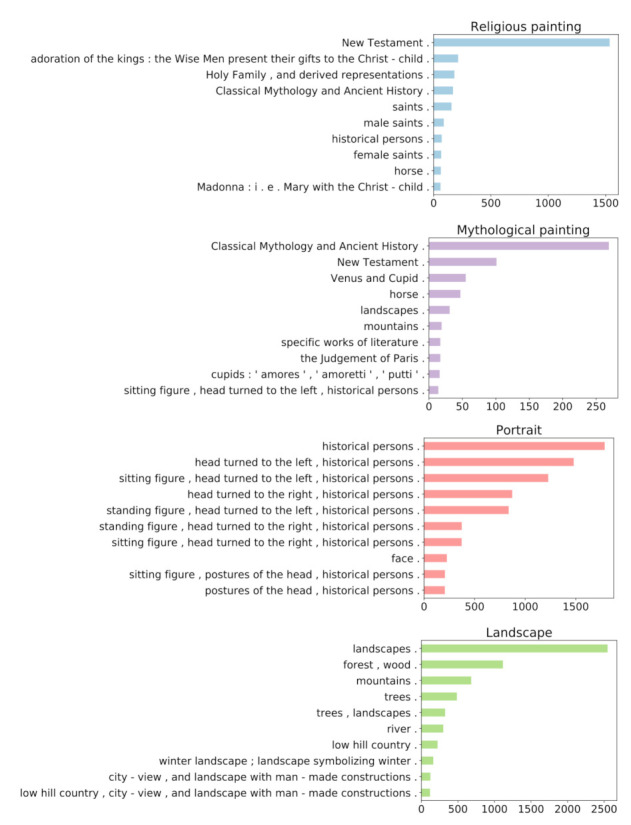
Distribution of most commonly generated captions in relation to four different genres in the WikiArt dataset.

The quality of the generated captions and the model’s capacity to generalize to new data are further explored by employing the model on another artwork dataset, a subset of the WikiArt dataset that includes labels related to the genre of the paintings. Figure 5 shows the distribution of the most commonly generated descriptions in relation to four different genre categories. From this basic analysis, it is obvious that the generated captions are meaningful in relation to the content and the genre categorization of images.

**Figure 6:**
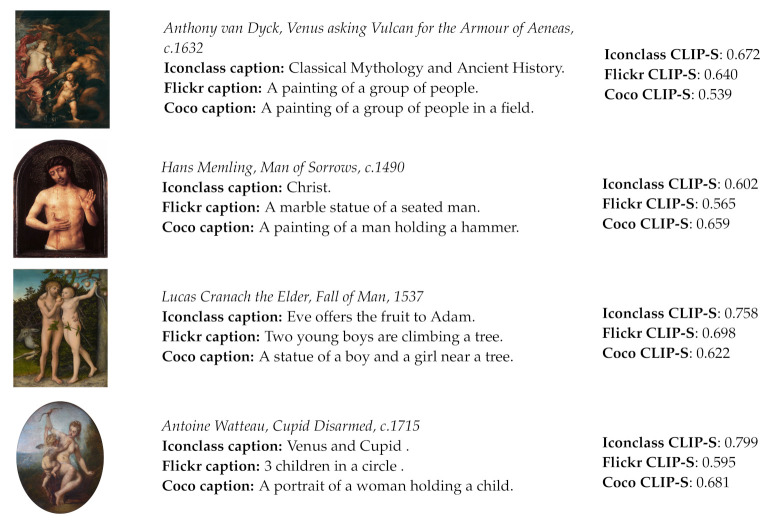
Examples from the WikiArt dataset with captions generated by models fine-tuned on the Iconclass, Flickr and COCO datasets.

To understand the contribution of the proposed model in the context of iconographic image captioning, it is interesting to compare the Iconclass captions with captions obtained from models trained on natural images. For this purpose, two models of the same architecture but fine-tuned on the Flickr 30 i MS COCO datasets were used. Figure 6 shows several examples from the WikiArt dataset with corresponding Iconclass, Flickr and COCO captions. It is evident that the other two models generate results that are meaningful in relation to the image content but do not necessarily contribute to producing more fine-grained and context-aware descriptions. However, the values of the CLIP-Score evaluation metric are, in general, higher for captions generated using the model pretrained on natural images than the Iconclass model.

The mean value of CLIP-S on the Iconclass captions of the WikiArt subset is 0.595, while the mean score of the Flickr caption is 0.684 and that of the Coco captions is 0.691. This result corresponds to the conclusion presented in [10], which suggests that, when assessing a direct description and a more non-literal caption, the CLIPScore will generally prefer the literal one. However, because the CLIP model is trained on an very large set of examples extracted from the internet, it has probably encountered some well-known cases of iconographic image-text relations in the training set. This explains the high values of the CLIPScore for the third and fourth examples in Figure 6.

**Figure 7:**
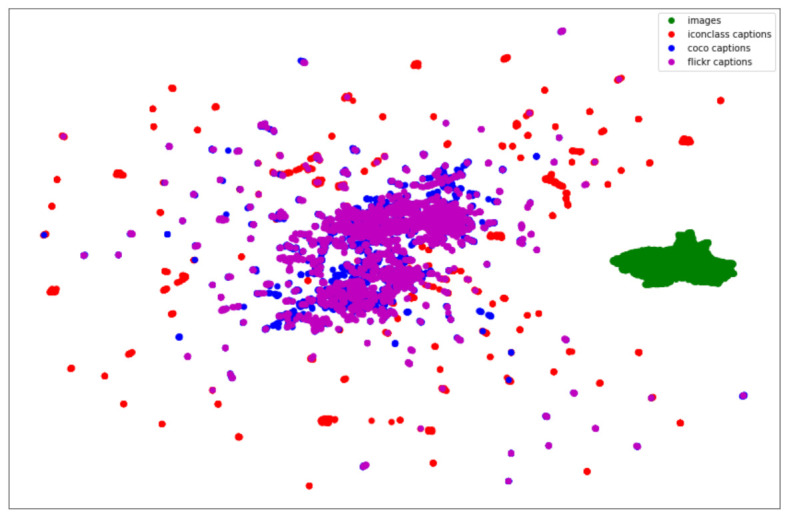
UMAP (Uniform Manifold Approximation and Projection) plot depicting the CLIP (Contrastive Language-Image Pre-Training) model embeddings of the images and various generated captions on a subset of the WikiArt dataset.

To gain a better understanding of the CLIPScore in relation to the various types of image captions and the images themselves, Figure 7 shows a projection of the image and different caption features obtained using the CLIP ViT-B/32 model.

The distribution of data points in Figure 7 indicates that the captions generated using the COCO and Flickr fine-tuned models are more aligned with each other, while the Iconclass captions are more dispersed. This is understandable considering the difference in the vocabulary and structure of the Iconclass descriptions. Overall, although the CLIPScore shows very good results in assessing the similarity of the image content and textual description, as well as particularly promising results in recognizing iconographic relations, it is still necessary to achieve a higher level of explainability of the CLIP model in order to determine its applicability for evaluating iconographic captions.

## 5. Conclusions

This paper introduces a novel model for generating iconographic image captions. This is achieved by utilizing a large-scale dataset of artwork images annotated with concepts from the Iconclass classification system designed for art and iconography. Within the scope of this work, the available annotations were processed into clean textual descriptions and the existing dataset was transformed into a collection of suitable image-text pairs. The dataset was used to fine-tune a transformer-based vision-language model. For this purpose, object classification aware region features were extracted from the images using the Faster R-CNN model. The base model in our fine-tuning experiment is an existing model, called the VLP model, that was pretrained on a natural image dataset on intermediate tasks with unsupervised learning objectives. Fine-tuning pretrained vision-language models represents the current state-of-the-art approach for many different multimodal tasks.

The captions generated by the fine-tuned models were evaluated using standard image captioning metrics and recently introduced reference-free metrics. Due to the specific properties of the Iconclass dataset, standard image captioning evaluation metrics are not very informative regarding the relevance and appropriateness of the generated captions in relation to the image content. The reference-free metric, CLIPScore, represents an interesting new approach for evaluating image captions based on the cosine distance between image and text embeddings from a joint feature space. This image captioning metric shows very promising results in evaluating the semantic relation of images and texts, particularly in the case of well-known iconographic image-text examples. However, it is still uncertain if it the best choice for assessing all iconographic image captions because it generally favours literal over non-literal image-text relations. In this context, one of the major directions for future research is related to exploring multimodal deep learning approaches in the context of non-literal relations between images and texts.

The overall quantitative and qualitative evaluations of the results suggest that it is possible to generate iconographically meaningful captions that capture not only the depicted objects but also the art historical context and relation between subjects. However, there is still room for significant improvement. In particular, the unbalanced distribution of themes and topics within the training set results in often wrongly identified subjects in the generated image descriptions. Furthermore, the generated textual descriptions are often very short and could serve more as labels rather than captions. Nevertheless, the current results show significant improvement in comparison to captions generated from artwork images using models trained on natural image caption datasets. Further improvement can potentially be achieved with fine-tuning the current model on a smaller dataset with more elaborate ground-truth iconographic captions.

## Figures and Tables

**Table 1:** Values of the evaluation metrics used for assessing the performance of the iconographic image captioning model on the Iconclass Caption test set. *CLIP-S and RefCLIP-S values are reported on a subset of the test set.

Evaluation Metric	Value (×100)
BLEU 1	14.8
BLEU 2	12.8
BLEU 3	11.3
BLEU 4	10.0
METEOR	11.7
ROUGE-L	31.9
CIDEr	172.1
CLIP-S *	59.67
RefCLIP-S *	68.35

**Table 2:** Spearman’s correlation coefficients between the values of standard and new metric scores on the Iconclass Caption test set (*p*-value < 0.001).

Standard Metric	Correlation with CLIP-S	Correlation with Ref CLIP-S
BLEU-1	0.355	0.686
BLEU-2	0.314	0.647
BLEU-3	0.281	0.629
BLEU-4	0.236	0.602
METEOR	0.315	0.669
ROUGE-L	0.298	0.647
CIDEr	0.315	0.656

## Data Availability

Additional material including the model weights and dataset are available at https://github.com/EvaCet/Iconclass-image-captioning.
